# Supplementary material for: Patterns of Intron Gain and Loss in Fungi
Source: PLoS Biol. 2004 Nov 30;2(12):e422. doi: 10.1371/journal.pbio.0020422 (PMC532390; doi:10.1371/journal.pbio.0020422)
Supplement: Table S1 — Also available at http://genes.mit.edu/NielsenEtAl/. (4.3 MB ZIP). [file pbio.0020422.st001.zip › NielsenEtAl/html/446.html]

AN5971.1.NCU05299.1.MG06030.1.FG01941.1


```
 CLUSTAL W (1.82) Multiple Sequence Alignments - Introns Inserted


Sequence 1: MG06030.1	243 aa
Sequence 2: FG01941.1	235 aa
Sequence 3: AN5971.1	239 aa
Sequence 4: NCU05299.1	273 aa
Alignment Length: 277 aa
Number Identitical Residues: 74 aa
Alignment Score (without introns) 3941


MG06030.1 	MRRSIQRLAS-----VKPQRYLEPGTATGLAGLRTHGSPRSTLLYLYSSTLEQLKAVPEH
NCU05299.1	MRAALRLLATATATVRPSARFLKPGSPTGLTGLGTHPSPRSALLYLYNHTLDKLKQIPEH
FG01941.1 	MRPTARVFA----------RYLEAGTPTGLTGLWTHATPRSTLLYLYGTTLNRLQKVPEN
AN5971.1  	MRATLRLLAS----VKP-ARYLEPFTPTGLTGLNTHPSPRPTLIYLYTSTLEKLKAFPES
          	** : : :*:         *:*:. :.***:** ** :**.:*:***  **::*: .** 

MG06030.1 	SVYRQSVEALTKHRMSILQSVVPPGHAEWAAKASKKIEANPDFFHSAAEGPVGGTSITRV
NCU05299.1	SLYRQSAEALTKHRLAIVEQYVPDGYDAWQERARKLLEKHK---SDLTARQFDGQHARLV
FG01941.1 	SMYRQSVEAVTKHRMSLVEQMVPPGYNEWAARAKELVSKNSSQFR-VSSGRIDGSEAHTV
AN5971.1  	SAYRQATEALTRHRLQIVESTKPAGYEAWLERVKKAVAAEP---ERFAS-------LRLA
          	* ***:.**:*:**: :::.  * *:  *  :. : :  .       :           .

MG06030.1 	DR-EGETFLVRKNQQQRDMRDEEWDGEPNEG--------PELEGARSDEERS---RQAKK
NCU05299.1	EGPDGRAYFIRQMVPPQDWRDVEWDGAVLDPHFSWVQTGEDVVGAVKLEDSDKLLELDKI
FG01941.1 	KL-GDKVFVVGRKHEASDVRIEEWDGEEDEG--------SEYEGIRTLKERE---DQATW
AN5971.1  	DG---SYAAIQRDDNSDNPRGEEWDGEKLEP---------TTEGPARTAEQQARWEKEIE
          	.        : :     : *  ****   :             *     : .        

MG06030.1 	MLRKSHDYSSR-----------------VDWEPEPQLTAEQ2VEEIENKLGAGLIEEVVQ
NCU05299.1	RESDPVAYRQGLRDLGIKMGGVVEDKSPVEWESEPPLSAEQ2IAEMEARIGSGLIEEVVQ
FG01941.1 	AERKPLEDHEK-----------------VEWEDEPQMTAEQ2VHELEQKIGAGLIEEIIQ
AN5971.1  	EATSSAADAK--SDFHTKQ---------MKWENEPALDADQ2IAEIEKKIGAGLIEEVIQ
          	   ..    .   .   .          :.** ** : *:* : *:* ::*:*****::*

MG06030.1 	VAEGELRLVKTMIEGKV2WESLEEQPAEGQWKYFERKAA
NCU05299.1	VAEGELKLVDIMTQARP2WEALEEEAPEGQWTYFERKE-
FG01941.1 	VAEGELKLVETMEKTKV2WEDLDEKPVPGQWTYFDRT--
AN5971.1  	VAEGELKLVDELYKSKS2WEELEEKPRPGQWTYFERKTD
          	******:**. : : :  ** *:*:.  ***.**:*.
```
